# High‐Density Lipoprotein‐Associated Cholesterol Abnormalities in a Clinical Outcomes Study of Dysferlin‐Deficient Limb–Girdle Muscular Dystrophy Type R2

**DOI:** 10.1002/jcsm.70042

**Published:** 2025-08-15

**Authors:** Zoe White, Laura Rufibach, Heather Gordish Dressman, Heather Hilsden, Dan Cox, Simone Spuler, John W. Day, Kristi J. Jones, Diana X. Bharucha‐Goebel, Emmanuelle Salort‐Campana, Alan Pestronk, Maggie C. Walter, Carmen Paradas, Tanya Stojkovic, Madoka Mori‐Yoshimura, Elena Bravver, Jordi Diaz‐Manera, Elena Pegoraro, Jerry R. Mendell, the Jain COS Consortium, Volker Straub, Pascal Bernatchez

**Affiliations:** ^1^ Department of Anesthesiology, Pharmacology & Therapeutics University of British Columbia (UBC) Vancouver Canada; ^2^ UBC Centre for Heart Lung Innovation St. Paul's Hospital Vancouver Canada; ^3^ Jain Foundation Seattle Washington USA; ^4^ Center for Translational Science, Division of Biostatistics and Study Methodology Children's National Health System Washington District of Columbia USA; ^5^ Pediatrics, Epidemiology and Biostatistics George Washington University Washington District of Columbia USA; ^6^ The John Walton Muscular Dystrophy Research Centre, Translational and Clinical Research Institute Newcastle University and Newcastle Hospitals NHS Foundation Trust Newcastle upon Tyne UK; ^7^ Charité Muscle Research Unit Experimental and Clinical Research Center, a Joint Cooperation of the Charité Medical Faculty and the Max Delbrück Center for Molecular Medicine Berlin Germany; ^8^ Department of Neurology and Neurological Sciences Stanford University School of Medicine Stanford California USA; ^9^ The Children's Hospital at Westmead The University of Sydney Sydney New South Wales Australia; ^10^ Department of Neurology Children's National Health System Washington District of Columbia USA; ^11^ National Institutes of Health (NINDS) Bethesda Maryland USA; ^12^ Neuromuscular and ALS Reference Center of Marseille La Timone University Hospital Marseille France; ^13^ Department of Neurology Washington University School of Medicine St. Louis Missouri USA; ^14^ Friedrich‐Baur‐Institute, Department of Neurology Ludwig Maximilian University of Munich Munich Germany; ^15^ Neuromuscular Unit, Department of Neurology Hospital U. Virgen del Rocío/Instituto de Biomedicina de Sevilla Sevilla Spain; ^16^ Centre de Référence des Maladies Neuromusculaires, Institut de Myologie, Sorbonne Université, Hôpital Pitié‐Salpêtrière APHP Paris France; ^17^ Department of Neurology, National Center Hospital National Center of Neurology and Psychiatry Tokyo Japan; ^18^ Neuroscience Institute, Carolinas Neuromuscular/ALS‐MDA Center Carolinas HealthCare System Charlotte North Carolina USA; ^19^ Department of Neuroscience University of Padova Padova Italy; ^20^ The Abigail Wexner Research Institute Nationwide Children's Hospital Columbus Ohio USA

**Keywords:** cholesterol, dysferlin, dyslipidemia, muscular dystrophy

## Abstract

**Background:**

Limb–girdle muscular dystrophy (MD) type R2 (LGMDR2, formerly LGMD2B) is an autosomal recessive form of MD caused by variants in the dysferlin gene, *DYSF*. It leads to slow proximal and distal muscle weakening that generally results in loss of ambulation around early adulthood but without the lethal cardiorespiratory dysfunction observed in the more severe Duchenne MD. How loss of dysferlin causes muscle fibre death is poorly understood, but recent evidence suggests a link between muscle wasting and loss of muscle cholesterol homeostasis with circulating lipoprotein abnormalities in many forms of MD.

**Methods:**

Cross‐sectional circulating total cholesterol (CHOL), high‐density lipoprotein‐associated cholesterol (HDL‐C), non‐HDL‐C, creatine kinase (CK), transaminase levels and bilirubin were collected as part of the Jain Clinical Outcomes Study of Dysferlinopathy, a large multicentre LGMDR2 patient cohort (*N* = 188), along with ambulatory function values.

**Results:**

We report that 43%, 49% and 50% of male patients were found to have abnormal circulating CHOL, HDL‐C and non‐HDL‐C levels, respectively, whereas in female patients 39%, 37% and 30% of values were in the abnormal range. Overall, 68% of the total cohort had at least one abnormal cholesterol value (78% of males and 60% of females) and 89% of male CHOL/HDL‐C ratios were in the suboptimal range (above 3.5). Although most patients were ambulant, the severity of circulating lipid abnormalities did not correlate with early loss of ambulation. Transaminase levels were lower in late‐stage LGMDR2 samples, whereas bilirubin remained unchanged, suggesting a low muscular mass rather than hepatic origin and the absence of major liver damage.

**Conclusions:**

Data from the largest natural history cohort of LGMDR2 patients support the concept that dyslipidemia is a comorbidity of LGMDR2, and the causal role of cholesterol abnormalities in muscle death should be further investigated.

## 1 Introduction

Limb–girdle muscular dystrophy type R2 (LGMDR2, formerly called LGMD2B) is an autosomal recessive form of muscular dystrophy (MD) caused by pathogenic variants in the *DYSF* gene, which leads to dysferlin deficiency [1, 2]. The main clinical phenotype associated with LGMDR2 is proximal muscle weakness, but dysferlin deficiency can also initially present with distal muscle weakness, which is often referred to as Miyoshi myopathy type 1 (MMD1), or a pseudometabolic phenotype characterised by isolated muscle pain during or after exercise [3]. However, despite this difference in initial presentation, natural history data show that these differing phenotypes are the same disease [4]. Although dysferlin is expressed in all muscle tissues, including the heart and diaphragm, as well as by many non–muscle cells [5, 6]. LGMDR2 progresses more slowly and does not lead to the terminal cardiopulmonary dysfunction typically seen in Duchenne muscular dystrophy (DMD). In mice, loss of dysferlin expression results in slow and mostly asymptomatic muscle loss [7], whereas in patients, early proximal and/or distal muscle weakness starts in the late teenage years and eventually progresses to affect both proximal and distal muscles with only minor changes in cardiorespiratory function with reduced forced vital capacity (< 90% of predicted value on average) and asymptomatic transatrial conduction anomalies [8]. Early work provided evidence that dysferlin can promote the fusion of resealing ‘patche’ following sarcolemma damage [7]; however, dysferlin has also been shown to be involved in T‐tubule structure and function [9, 10], vesicle trafficking [6], endocytosis [11] and lipid metabolism [12, 13, 14]. Nevertheless, which one of these functions plays the most prominent role in the disease pathogenesis has yet to be determined. Dysferlin was initially thought to mostly reside at the sarcolemma despite not being part of the dystrophin–glycoprotein‐associated complex [7], whereas more recent evidence has shown it is also highly localised to the muscle T‐tubules, the site of which cholesterol [15] and Ca^2+^ tightly regulate muscle excitation–contraction coupling [9].

Recently, we reported that elevated circulating non‐high‐density lipoprotein‐associated cholesterol (non‐HDL‐C) can unexpectedly exacerbate the mild phenotype of dysferlin‐null mice [16], resulting in severe muscle death and replacement with large fibroadipose lesions and early loss of ambulation. This prompted us to investigate cholesterol homeostasis in dysferlin‐deficient mice and patients, where we found evidence of high free muscle cholesterol (FC) accumulation in mice as well as low high‐density lipoprotein‐associated cholesterol (HDL‐C) in patients [17]. Taken together, these data suggested major cholesterol abnormalities in the absence of dysferlin that could play a causal role in the muscle degenerative process. Herein, rather than use biobanked samples, we investigated circulating lipids and markers of hepatic and muscle values from the Jain Foundation's International Clinical Outcome Study (COS) for dysferlinopathy, a multicentre study aimed at characterising the natural history of LGMDR2 in 203 patients across 15 sites in eight countries (Europe, the United States, Japan and Australia) [18]. COS collected clinical and biochemical data at remote sites to build a complete picture of dysferlinopathy and how it progresses over time. We report a high prevalence of abnormal circulating lipid values in this population and early transaminase elevation with only minor changes in alkaline phosphatase (ALP), suggesting the likely presence of a dyslipidaemic state that is not the result of liver dysfunction. These findings along with evidence of a high prevalence of dyslipidaemia in DMD and exacerbation of mild MD rodent phenotypes through hypercholesterolaemia and thermoneutrality [19, 20, 21] support the concept that many forms of MD may interfere with cholesterol homeostasis to cause muscle wasting [22]. Dysferlin deficiency likely leads to a new form of dyslipidaemia as a metabolic comorbidity that may contribute to skeletal muscle degeneration.

## 2 Subjects/Materials and Methods

### 2.1 Human Samples and Serology

Dysferlinopathy patient data included in this study were retrospectively obtained from the Clinical Outcome Study for Dysferlinopathy (COS) steering committee. All study participants provided informed consent. The study was approved by ethical review boards at each site. Blood sampling from nonfasting COS participants was performed at 14 COS‐approved sites across eight countries: the United States (Saint Louis MO, Columbus OH, Charlotte NC and Stanford CA); Europe (Germany [Munich and Berlin]); Italy (Padova); France (Paris and Marseilles); Spain (Barcelona and Sevilla); the United Kingdom (Newcastle); Australia (Sydney); and Japan (Tokyo).

Whole blood was centrifuged at 3000 rpm/1500 g for 10 min followed by routine serology testing, and value collection was done by the John Walton Muscular Dystrophy Research Centre at Newcastle University. Patient lipid levels were plotted relative to the 2018 American College of Cardiology/American Association Task Force on Clinical Practice Guidelines as previously reported [21, 23], the American Heart Association [24] and Nantsupawat et al. [25]. Nonfasting lipoprotein ranges for patients ≤ 19 and ≥ 20 years are listed in Table 1, as are abnormal serum ranges for ALT, AST, ALP, total bilirubin and CK [26, 27]. Of note, lipoprotein levels are shown to change minimally in response to food intake (triglycerides show the most variation, although at maximum 20% or ±0.3 mmol/L) [28, 29].

**TABLE 1:** Above normal nonfasting reference values for blood cholesterol, liver enzymes and creatine kinase.

	≤ 19 years	≥ 20 years	
	Male and female	Male	Female
TC	> 4.4 mmol/L	> 5.2 mmol/L	> 5.2 mmol/L
Non‐HDL‐C	> 3.1 mmol/L	> 4.1 mmol/L	> 4.1 mmol/L
HDL‐C	< 1.16 mmol/L	< 1.03 mmol/L	< 1.3 mmol/L
TC/HDL‐C	> 3.5	> 3.5	> 3.5
ALT	> 40 U/L		
AST	> 37 U/L		
ALP	> 110 U/L		
Total bilirubin	> 17 μmol/L		
CK	> 250 U/L		

### 2.2 Statistical Analyses

Statistical analyses are described in the figure legends. Data were analysed using GraphPad PRISM v6. Data are mean ± SEM. Results with *p* values of less than 0.05 were considered statistically significant.

## 3 Results

### 3.1 Plasma Lipoprotein Markers in Dysferlinopathy Patients

Serology values for total cholesterol (CHOL), high‐density lipoprotein cholesterol (HDL‐C), alanine transaminase (ALT), aspartate aminotransferase (AST), ALP and total bilirubin were retrospectively obtained from the COS patient cohort as part of the study protocol. Results were entered into the study database and supplied with approval from the Jain COS steering committee. There were initially 203 patients enrolled in the study. Of these, 197 had prereported CHOL values, and 171 had values for HDL‐C collected at each clinical COS site. Patient data was excluded from nine patients on medications shown to have profound regulatory effects on plasma lipoprotein readouts: Two were on HMGCoA–reductase inhibitors (simvastatin or atorvastatin), three on the cholesterol absorption blocker ezetimibe and four on the corticosteroid prednisone. Lipid data were not clinically reported for six patients for either medical or personal reasons. Thus, 188 CHOL and 162 HDL‐C patient values were included in the final analysis. Calculations of non‐HDL‐C (which includes LDL‐C, VLDL‐C, IDL‐C and chylomicrons quantities) and CHOL/HDL‐C ratios (a typical readout of cardiovascular disease risk) were additionally performed for patients with values for both CHOL and HDL‐C. Any cholesterol values provided in mg/dL were converted to mmol/L (value multiplied by 0.02586). For bilirubin, values provided in mg/dl were converted to μmol/L (value multiplied by 17.1). For analysis, patients were stratified into the following age groups: < 19, 20–29, 30–39, 40–49 and > 50 years. Mean ages are reported for both male and female cohorts in Table 2.

**TABLE 2:** Patient data for Clinical Outcome Study (COS) enrolled dysferlinopathy patients with site‐collected lipoprotein measures (*N* = 188 patients).

	Mean age (years)	
	Male	Female
≤ 19 years	14.6 ± 1.6 (5)	15.8 ± 2.0 (5)
20–29 years	25.8 ± 0.6 (23)	25 ± 0.6 (20)
30–39 years	33.9 ± 0.6 (24)	34.0 ± 0.5 (30)
40–49 years	44 ± 0.5 (24)	44.8 ± 0.65 (22)
> 50 years	55 ± 1.9 (10)	57.6 ± 1.6 (25)

*Note:* Data are mean ± SEM. In parentheses (the number of patients in each age group).

**FIGURE 1:**
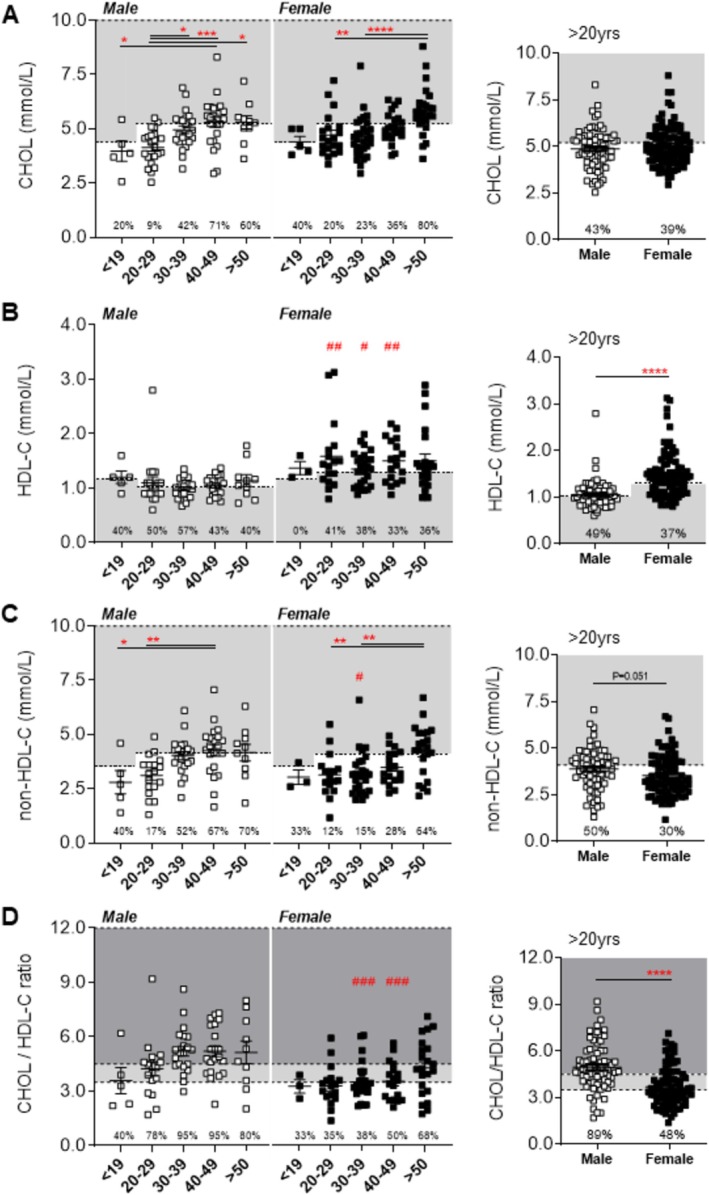
Baseline measures of plasma lipoprotein levels in male and female LGMDR2 patients stratified by age. (A–D) Scatter plots of LGMD2B lipoprotein plasma lipoprotein levels (total cholesterol [CHOL], high‐density lipoprotein cholesterol (HDL‐C), calculated non‐HDL‐C and CHOL/HDL‐C ratios that fall within and outside of normal levels for paediatric (≤ 19 years) and adult (20 to > 50 years) male and female LGMDR2 patients. Combined lipoprotein data for all patients are also demonstrated for each sex for each parameter. Two‐way ANOVA with Tukey's post hoc tests were used for direct comparisons between ages specific to each sex; (*) *p* < 0.05; (**) *p* < 0.01; (***) *p* < 0.001; (****) *p* < 0.0001. Two‐way ANOVA with Sidak's post hoc tests were used for direct mean comparisons between male and female values; (#) *p* < 0.05; (###) *p* < 0.001; (####) *p* < 0.0001. For combined data, a Student *t*‐test was used for direct mean comparisons between male and female values; (*) *p* < 0.05; (****) *p* < 0.0001. Light grey zone denotes abnormal ranges for CHOL, HDL‐C and non‐HDL‐C. The number of patients falling outside of normal range (abnormal values) is listed as a percentage within the graph. For CHOL/HDL‐C ratios, light grey indicates above optimal ranges, and dark grey indicates abnormal. The number of patients falling outside of the optimal range is listed as a percentage within the graph.

Data from dysferlinopathy patients showed that plasma CHOL levels increased by 28% in male and 23% in female patients aged between the 20‐ to 29‐year and > 50‐year cohorts, respectively, with no significant differences in total CHOL levels reported between the sexes (Figure 1A). A higher prevalence of above normal plasma CHOL concentrations (≥ 60%) was observed in male patients ≥ 40 years, whereas greater abnormalities were observed in female patients ≥ 50 years (Figure 1A). Overall, in adults > 20 years, 43% of male and 39% of female LGMD2R patients displayed abnormal plasma CHOL levels, with combined averages of 4.87 ± 0.12 and 5.04 ± 0.11 mmol/L, respectively **(**Figure 1A). From a global perspective, CHOL levels in LGMD2R patients were comparable to age‐standardised means reported for Central/Eastern European (4.98 ± 0.1 mmol/L and 5.0 ± 0.11 mmol/L) and high‐income Western countries (4.71 ± 0.08 mmol/L and 4.82 ± 0.08 mmol/L) (2018) [30, 31] Moreover, previously published CHOL levels in serum from LGMD2R COS study patients were shown to be similar to healthy age‐matched controls, for both sexes [17]. In contrast, no age‐associated increases in plasma HDL‐C concentrations were observed in either male or female cohorts, although females had higher plasma HDL‐C levels than males across the following age groups: 20–29 years (by 43.6%), 30–39 years (by 31%) and 40–49 years (by 44%), as well as overall (*p* < 0.0001) (Figure 1B). Males, however, displayed a higher overall prevalence of low HDL‐C than females (49% vs. 37%, respectively [Figure 1B]), whereas in historical data from US adults, only ∼18.0% (28.5% of men and 8.9% of women) have low plasma HDL‐C [30]. Herein, LGMD2R patients present with overall HDL‐C averages of 1.06 ± 0.035 and 1.47 ± 0.05 mmol/L in males and females, which when compared to our previously published data in COS patient serum, was significantly less than age‐matched controls, despite having similar rates of detected abnormalities [17]. Analysis of non‐HDL‐C levels, which factor in the LDL‐C, VLDL‐C and chylomicron contributions, showed increases of 53% in male and 34% in female patients aged between < 19 and 40–49 years and 20‐ to 29‐ and > 50 years, respectively (*p* < 0.05), with a higher prevalence of abnormalities observed in male patients over the age of 30 years and females > 50 years (Figure 1C). Overall, in adults > 20 years, males were shown to have higher plasma non‐HDL‐C levels (3.88 ± 0.13 mmol/L) than females (3.56 ± 0.11 mmol/L; *p* = 0.051; Figure 1C). Finally, analysis of CHOL/HDL‐C ratios, an indicator of cardiovascular disease risk, demonstrated that LGMD2R males had a higher prevalence of suboptimal (> 3.5 mmol/L) and abnormal (> 4.5 mmol/L) [32] and earlier commencement of CHOL/HDL‐C anomalies compared to female patients (89% vs. 48%; 4.95 + 0.18 mmol/L vs. 3.67 + 0.14 mmol/L, respectively, Figure 1D), which was again confirmed in LGMD2R COS study serum samples relative to control patients [17].

**FIGURE 2:**
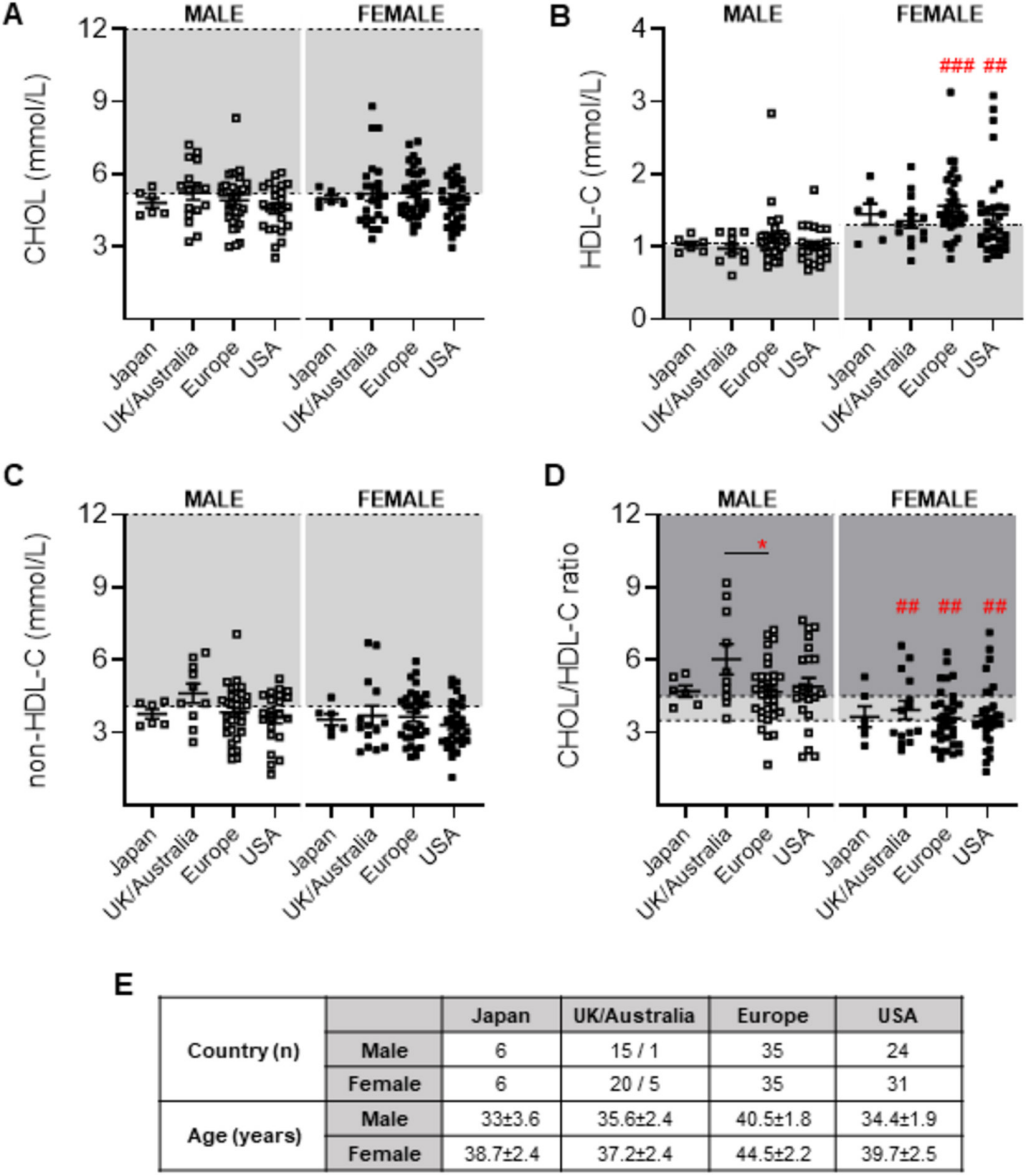
Baseline measures of plasma lipoprotein levels in male and female LGMDR2 patients stratified by region. (A–D) Scatter plots of LGMDR2 lipoprotein plasma lipoprotein levels (Total Cholesterol (CHOL), high‐density lipoprotein cholesterol (HDL‐C), calculated non‐HDL‐C and CHOL/HDL‐C ratios that fall within and outside of normal levels for adult (20 to > 50 years) male and female LGMD2B patients from Japan, the United Kingdom/Australia, Europe and the United States. (E) Number of patients from each country and average age ± SEM. Two‐way ANOVA with Tukey's post hoc tests were used for direct comparisons between ages specific to each sex; no significance detected. Two‐way ANOVA with Sidak's post hoc tests were used for direct mean comparisons between male and female values; (#) *p* < 0.05; (###). Light grey zone denotes abnormal ranges for CHOL, HDL‐C and non‐HDL‐C. The number of patients falling outside of normal range (abnormal values) is listed as a percentage within the graph. For CHOL/HDL‐C ratios, light grey indicates above optimal ranges, and dark grey indicates abnormal. The number of patients falling outside of the optimal range is listed as a percentage within the graph. No differences in age were detected via two‐way ANOVA for Panel E.

Given that global dietary and lifestyle patterns can also influence plasma cholesterol levels, patients were further stratified into the following geographical locations, and CHOL, HDL‐C, CHOL/HDL‐C ratios and non‐HDL‐C were analysed: (1) Japan (Tokyo); (2) the United Kingdom/Australia (Newcastle/Sydney); (3) Europe (Germany, Italy, France and Spain); and (4) North America (the United States). No significant differences in CHOL, HDL‐C or non‐HDL‐C were observed across the four regional areas in either male or female subpopulations (Figure 2A–D), although CHOL/HDL‐C ratios between males from Australia/the United Kingdom were significantly higher than males from Europe (*p* < 0.05). Higher HDL‐C levels were observed in females from Europe and the United States, whereas lower CHOL/HDL‐C ratios were observed in female patients from the United Kingdom/Australia, Europe and the United States, compared to males (Figure 2B,D **)**. The number of patients from each region was included in Figure 2E, and patient ages were similar between all analysed groups.

**FIGURE 3:**
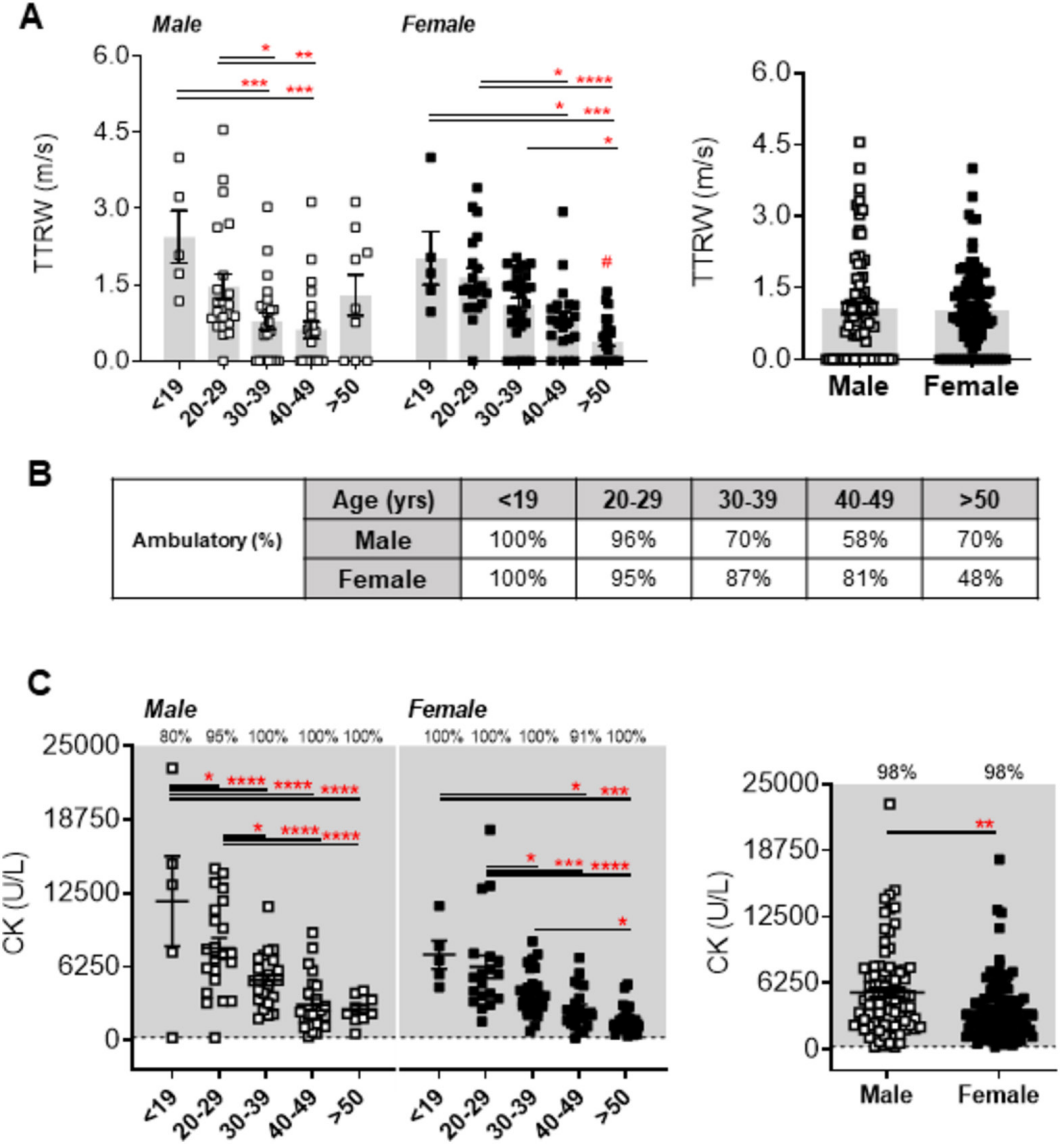
Baseline measures of ambulatory function and enzymatic markers of muscle wasting in male and female LGMDR2 patients stratified by age. (A) Velocity/speed (m/s) measures for the time to run/walk 10 m test TTRW test for paediatric (≤ 19 years) and adult (20 to > 50 years) male and female LGMDR2 patients. Combined TTRW data for all patients is also demonstrated for each sex. (B) Percentage (%) of male and female patients clinically reported as “Ambulatory” stratified by age. (C) Creatine kinase for paediatric (≤ 19 years) and adult (20 to > 50 years) male and female LGMD2B patients. Two‐way ANOVA with Tukey's post hoc tests were used for direct comparisons between ages specific to each sex; (*) *p* < 0.05; (**) *p* < 0.01; (***) *p* < 0.001; (****) *p* < 0.0001. Two‐way ANOVA with Sidak's post hoc tests were used for direct mean comparisons between male and female values; (#) *p* < 0.05. In Panel C: Grey zone denotes abnormal ranges for each specific parameter. The number of patients falling outside of normal range (abnormal values) is listed as a percentage within the graph.

We next attempted to link dyslipidemia in LGMDR2 patients to the loss of ambulation by analysing TTRW scores (time to run/walk 10 m). As TTRW scores were only obtained from ambulant patients, any patient listed as non‐ambulatory or unable to walk 10 m without usual orthotics or walking aids was assigned a velocity or speed value of ‘0 m/s’ and included in the analysis. In male patients with LGMDR2, incremental decreases in walking speed were observed between < 19 and 40–49 years of age in males (by 46%; *p* < 0.05; Figure 3A). Similar decreases in walking speed (by 80%; *p* < 0.0001; Figure 3A) were observed in female patients starting from < 19 years. Moreover, females > 50 years had significantly slower walking speeds than males of the same age (by 77%; *p* < 0.0001; Figure 3A), with only 48% of females being classified as ambulatory at this age (Figure 3B). Furthermore, serum creatine kinase (CK), a diagnostic marker for muscle damage, was significantly decreased in male (by 78%) and female (by 76%) patients between < 19 and 50 years (*p* < 0.0001; Figure 3C), with over 98% of all patients displaying abnormal levels.

**FIGURE 4:**
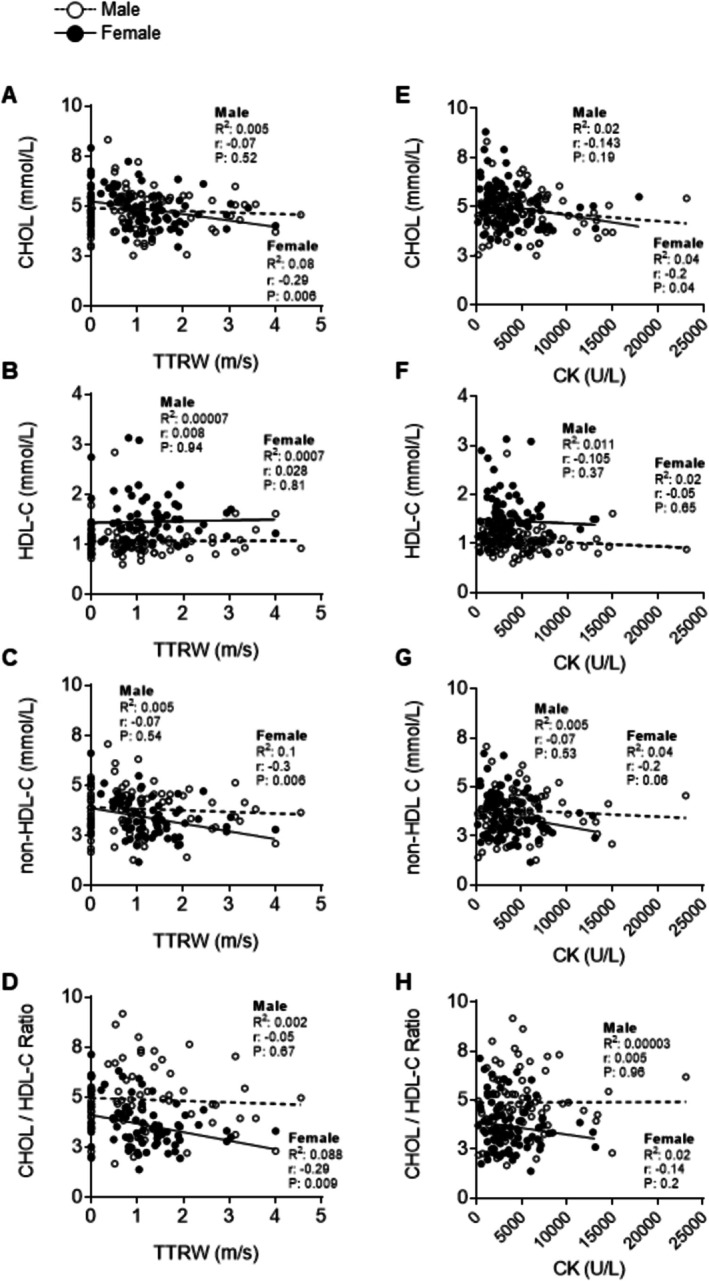
Correlation between plasma lipoprotein levels, ambulatory function and CK levels in male and female LGMDR2 patients at baseline. (A–D) Total Cholesterol (CHOL), high‐density lipoprotein cholesterol (HDL‐C), calculated non‐HDL‐C and CHOL/HDL‐C ratio plotted against the velocity/speed (m/s) measures for the time to run/walk 10 m test (TTRW). (E–H) Total cholesterol (CHOL), high‐density lipoprotein cholesterol (HDL‐C), calculated non‐HDL‐C and CHOL/HDL‐C ratio plotted against the creatine kinase (CK) levels (U/L). Pearson correlation was used to report *r*, *R* ^2^ and *p* value for male and female datasets for each parameter.

Because rodent studies have demonstrated that aberrant cholesterol metabolism can accelerate LGMDR2‐associated muscle fibre death in preclinical models [16, 33], plasma CHOL, HDL‐C, CHOL/HDL ratios, and non‐HDL‐C levels were correlated with both TTRW (m/s) and CK (Figure 4A–H). Interestingly, HDL‐C was not significantly correlated with ambulatory dysfunction in either male or female patients (Figure 4B). Although in females, CHOL (*p* = 0.006), CHOL/HDL‐C ratios (*p* = 0.009) and non‐HDL‐C levels (*p* = 0.006) were shown to be significant but weakly correlated with TTRW (m/s) in patients with LGMDR2 (Figure 4A,C,D). In contrast, cholesterol values were less associated with plasma CK, whereby only CHOL (*p* = 0.04) was significantly but weakly correlated in female patients (Figure 4E–H).

**FIGURE 5:**
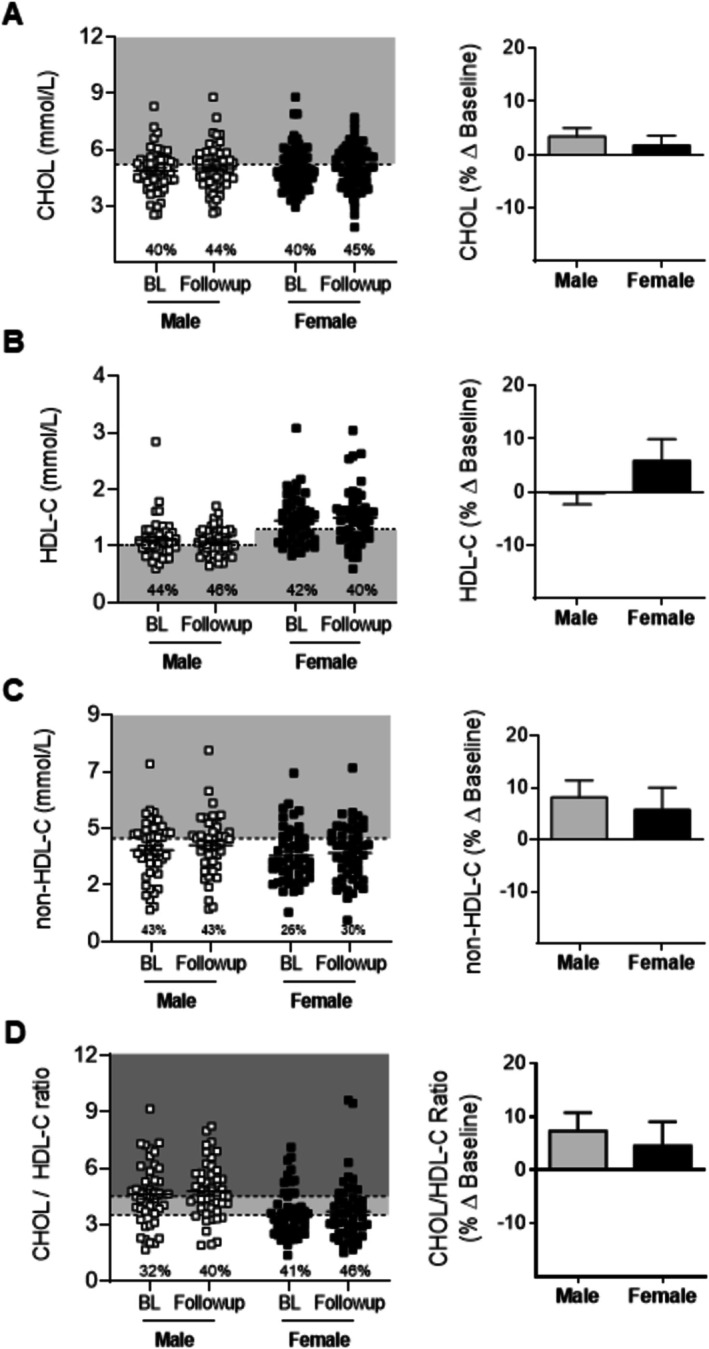
Plasma lipoprotein levels in male and female LGMDR2 patients with both baseline (BL) and 36‐month follow‐up measures. (A–D) Scatter plots of LGMDR2 lipoprotein plasma lipoprotein levels for total cholesterol (CHOL), high‐density lipoprotein cholesterol (HDL‐C), calculated non‐HDL‐C and CHOL/HDL‐C ratios that fall within and outside of normal levels for adult (20 to > 50 years) male and female LGMDR2 patients. Two‐way repeated measures ANOVA with Sidak's post hoc tests were used for direct comparisons between baseline and follow‐up values for males and females. The percentage change (Δ) from baseline for each patient has also been subsequently graphed. Light grey zone denotes abnormal ranges for CHOL, HDL‐C and non‐HDL‐C. The number of patients falling outside of normal range (abnormal values) is listed as a percentage within the graph. For CHOL/HDL‐C ratios, light grey indicates above optimal ranges, and dark grey indicates abnormal. The number of patients falling outside of the optimal range is listed as a percentage within the graph.

Of the 188 patients with CHOL values, 57 males and 75 females also had a longitudinal follow‐up measurement taken 36 months postbaseline (*N* = 132 total). Overall, CHOL, HDL‐C, CHOL/HDL‐C ratios and non‐HDL‐C values were similar between baseline and follow‐up measures in both males and females, with longitudinal changes for each parameter not exceeding 10% (Figure 5A–D).

### 3.2 Markers of Liver Dysfunction in Plasma of Patients With LGMDR2

**FIGURE 6:**
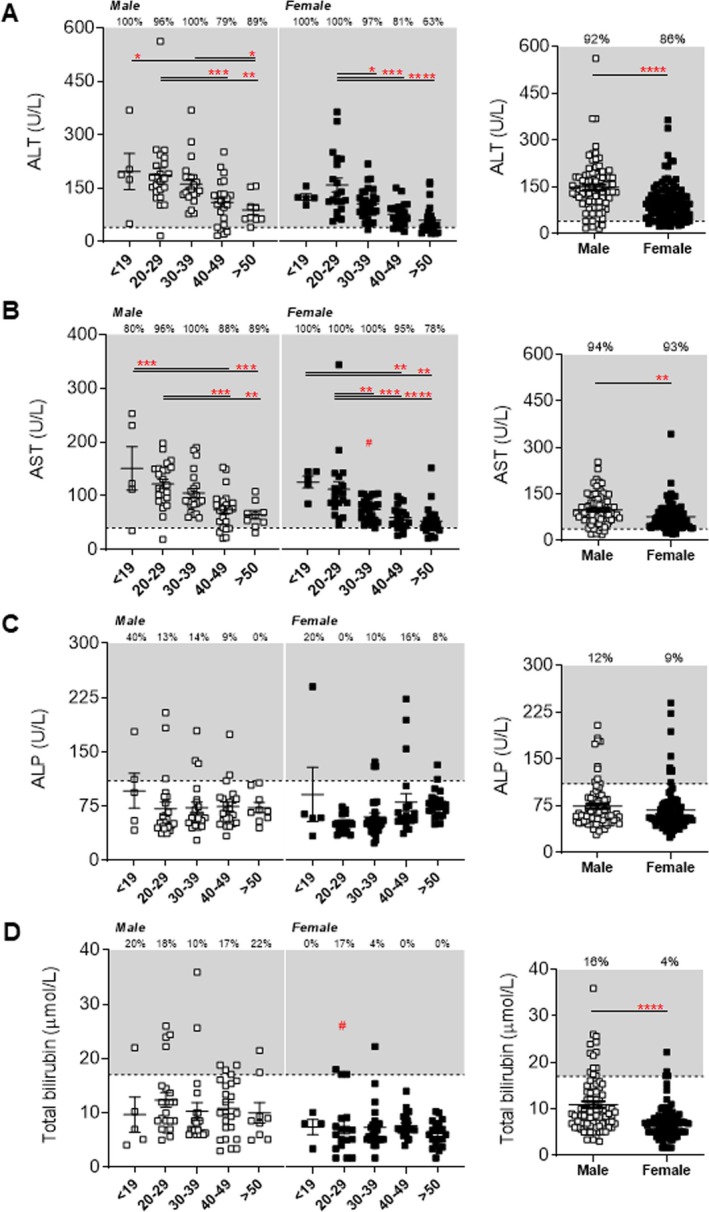
Clinical markers of liver dysfunction in male and female LGMDR2 patients stratified for age (at baseline). (A–D) Scatter plots of LGMDR2 alanine transaminase (ALT), aspartate aminotransferase (AST), alkaline phosphatase (ALP) and bilirubin levels that fall within and outside of normal levels for paediatric (≤ 19 years) and adult (20 to > 50 years) male and female LGMDR2 patients. Combined data for all patients is also demonstrated for each sex for each parameter. Two‐way ANOVA with Tukey's post hoc tests were used for direct comparisons between ages specific to each sex; (*) *p* < 0.05; (**) *p* < 0.01; (***) *p* < 0.001; (****) *p* < 0.0001. Two‐way ANOVA with Sidak's post hoc tests were used for direct mean comparisons between male and female values; (#) *p* < 0.05. For combined data, a Student *t*‐test was used for direct mean comparisons between male and female values; (*) *p* < 0.05; (**); *p* < 0.01; (***) *p* < 0.001; (****) *p* < 0.0001. Grey zone denotes abnormal ranges for each specific parameter. The number of patients falling outside of normal range (abnormal values) is listed as a percentage within the graph.

Plasma alanine aminotransferase (ALT) and aspartate transaminase (AST) were examined as they are classical markers of hepatocellular injury, which is relevant as the liver is the main regulator of lipoprotein metabolism. However, in the context of LGMDR2, transaminases are mostly released by muscle cells in response to damage [26] (Figure 6A,B). As expected, abnormal elevations of ALT and AST were observed in the plasma of both males and females across all age groups and decreased with age, similarly to CK, with > 86% of all patients presenting with above normal clinical levels (Figure 6A,B). In males, age‐associated decreases were also observed between < 19 and > 50 years (55% and 57% for ALT and AST, respectively; *p* < 0.05), whereas, in females, reduced ALT and AST were observed between 20 and 29 years and > 50 years (62%; *p* < 0.0001) and < 19 and > 50 years, respectively (56%; *p* < 001) (Figure 6A,B). Overall, female dysferlinopathy patients displayed lower ALT (by 32%; *p* < 0.0001) and AST (by 22%; *p* < 0.001) levels than males (Figure 6A,B). Additional retrospective analysis of ALP and bilirubin, two broad clinical markers of obstructive liver abnormalities, revealed mostly unaffected levels by age in either sex, and overall abnormalities were detected in only 4%–16% of the total patient population (Figure 6C,D). Although GGT was not collected from the plasma of patients in this retrospective cohort, significantly lower serum GGT values in COS study patients were reported elevated (but within normal clinical ranges), compared to healthy, age‐matched controls [17].

## 4 Discussion

Compared to the more common and severe form of DMD, the low prevalence and slowly progressing phenotype of LGMDR2 hampers research on how loss of dysferlin expression interferes with skeletal muscle integrity. The current work, based on data from the largest natural history study of dysferlinopathy, suggests that abnormal cholesterol metabolism could be considered as a metabolic comorbidity in patients with dysferlin deficiency [17]. Having provided evidence in dysferlin‐null animals that high CHOL/HDL‐C ratios drastically exacerbate muscle wasting, an effect prevented with cholesterol absorption inhibitor ezetimibe [16, 33], it is tempting to speculate that the HDL‐C lipoprotein abnormalities we report in patients with LGMDR2 may be implicated in the process of skeletal muscle degeneration [33]. Notably, past work from our group has documented low HDL‐C in complementary biobanked serum from LGMD2R COS study samples, and DMD subjects compared to healthy controls [17, 21, 34]. Although the values collected in our natural history cohort did not include TG and LDL‐C, which is a key limitation, it must be noted however that dyslipidemia is estimated to be as high as 39% in the world's adult population [35], whereas data from the US Centre for Disease Control reveal that 26.6% of men and 8.5% of women had low HDL‐C between 2015 and 2018—lower than the prevalence in LGMDR2 our analysis revealed. However, we acknowledge that healthy control samples are required for future studies to further assess the prevalence rates of dyslipidemia in LGMD2R patient populations. Approaches that modulate cholesterol metabolism should be carefully tested in this group of patients, as statins and their unique ability to inhibit muscle cholesterol synthesis have been shown to attenuate disease severity in various models of MD [33, 36].

From a cardiovascular disease perspective, current data suggest that the lipid profile of dysferlinopathy patients is associated with a higher risk for cardiovascular disease due to lower HDL‐C and elevated CHOL/HDL‐C [17]. Dysferlin‐null mice did not show increased atherosclerosis when on an ApoE‐deficient background, despite extensive muscle cell death [34], whereas dysferlin‐deficient patients show no major differences in early cardiorespiratory outcomes [8]. As lipid profiles associated with atherosclerosis drastically exacerbate dysferlinopathy and DMD in rodents, a better understanding of the origin of dyslipidemia and how to attenuate its deleterious effect on MD‐associated muscle degeneration is relevant to patient care. Few gene mutations have been linked to low HDL‐C; these include ABCA1, ANGPTL4 and LCAT mutations [37], which are critical for reverse cholesterol transport, as nascent HDL particles are released from the liver to collect excess cholesterol from remote cells. As dysferlin deficiency causes HMGCoAR dysregulation and free cholesterol (FC) accumulation in rodent muscle [17], it is tempting to speculate that there is also insufficient reverse FC transport from either affected muscle tissues or myofibers in part due to low circulating HDL‐C. Another possibility is that dysferlin can facilitate the repair of damaged membranes through the fusion of lysosomes, a key cholesterol‐regulating organelle [38], through acid sphingomyelinase, which is linked to the Niemann–Pick group of lysosomal storage diseases [39]. We found no such abnormalities in dysferlin‐deficient liver samples [17], which may suggest that circulating cholesterol abnormalities are of muscle rather than liver origin. Despite the absence of HMGCoAR and FC changes in dysferlin‐deficient liver samples, the dynamic interplay between muscle and liver homeostasis cannot be underestimated. Liver regeneration is known to be attenuated in settings of sarcopenia‐associated muscle atrophy [40], whereas healthy skeletal muscle typically represents 40% of the total body mass, which can influence circulating lipoprotein profiles.

Individuals with dysferlinopathy are afflicted by far fewer comorbidities than their DMD counterparts for multiple reasons; the steroids DMD patients are managed with often cause obesity and major changes in adipose tissue metabolism in addition to intramuscular fat accumulation, whereas steroid‐naïve patients with DMD show no or little increases in perivisceral or subcutaneous fat mass [41, 42, 43, 44]. Steroid therapy also affects bone growth and puberty, whereas loss of dystrophin activity in brain cells causes a range of neurological symptoms of DMD. In contrast, dysferlinopathy is not managed with steroids and, as expected, does not cause obesity despite loss of mobility, with little cardiorespiratory symptoms. Of note, the decline in activity observed in LGMD2R could be behind the reduction in HDL‐C we report, as physical activity is well known to increase HDL‐C. To err on the side of caution, we have documented that despite showing steeper declines in ambulation, DMD patients have HDL‐C levels comparable to less severe Becker MD patients, whereas DMD canine samples showed higher plasma HDL‐C than normal and carrier controls, suggesting that low MD‐associated exercise does not necessarily reduce HDL‐C [21]. In the COS cohort, dysferlinopathy does not appear to be associated with cardiac comorbidities or symptoms besides minor arrhythmias, whereas changes in lung function and dyspnoea have been documented [8]. Vascular abnormalities such as hypertension and leg swelling were reported, but the former was not more common than in the general population, whereas the latter did not appear to be related to cardiac function and is well known to occur in cases of reduced mobility. This may imply a more primary type of dyslipidaemia linked to muscle degeneration rather than secondary dyslipidaemia induced by pharmacotherapy or liver dysfunction. The novel concept that LGMDR2 and other MDs represent a new class of genetic dyslipidaemias remains to be confirmed [22]. This could help rationalise why statins cause myopathies in settings of cardiovascular disease prevention, as cholesterol dysregulation in skeletal muscle tissues may be akin to an Achilles' heel [45, 46].

## Author Contributions

All authors designed the study and edited the manuscript. Zoe White performed data analyses.

## Conflicts of Interest

The authors declare no conflicts of interest.
